# Processed pseudogene insertions in somatic cells

**DOI:** 10.1186/1759-8753-5-20

**Published:** 2014-07-02

**Authors:** Haig H Kazazian

**Affiliations:** 1 Institute for Genetic Medicine, Johns Hopkins University School of Medicine, Baltimore, MD 21205, USA

**Keywords:** Processed pseudogenes, L1 retrotransposons, Polymorphism, Cancer, Chronic granulomatous disease

## Abstract

Processed pseudogenes are copies of messenger RNAs that have been reverse transcribed into DNA and inserted into the genome using the enzymatic activities of active L1 elements. Processed pseudogenes generally lack introns, end in a 3’ poly A, and are flanked by target site duplications. Until recently, very few polymorphic processed pseudogenes had been discovered in mammalian genomes. Now several studies have found a number of polymorphic processed pseudogenes in humans. Moreover, processed pseudogenes can occur in somatic cells, including in various cancers and in early fetal development. One recent somatic insertion of a processed pseudogene has caused a Mendelian X-linked disease, chronic granulomatous disease.

## Background

Pseudogenes are sequences present in essentially all animal genomes that have many characteristics of genes, but are defective for production of protein. Of course, like most definitions that are 30 years old and based on incomplete information, this one has also been modified. We now know of many pseudogenes that are active in making proteins. Of the more than 14,000 pseudogenes in the human genome [1], at least 10% are no longer ‘pseudogenes’ and are active [1,2]. Many active ‘pseudogenes’ are gene duplicates that contain introns and are situated in close proximity to their active gene copies. These gene duplicates make up one class of pseudogenes. An interesting example of a duplicate pseudogene is the φζ gene in the α-globin gene cluster [3]. This pseudogene has only six nucleotide differences from its parent ζ (zeta) gene, and one of these differences leads to a nonsense codon. In eight populations studied, the nonsense codon is corrected by gene conversion in 15% to 50% of α-globin gene clusters. However, RNA emanating from the corrected φζ gene could not be detected [3].

Although there are many duplicate pseudogenes in the human genome, the majority of human pseudogenes, more than 7,800 [1], belong to the second class, and are called processed pseudogenes (PPs). The term processed pseudogene was first proposed in 1977 to describe a sequence of a 5S gene of *Xenopus laevis*[4]. PPs are found in the genomes of many animal species [2] and have the following characteristics: 1) their sequences are very similar to the transcribed portion of the parent gene; 2) they lack all or most introns, so they appear to be cDNA copies of processed mRNAs; 3) they have a poly A tail attached to the 3’-most transcribed nucleotide; and 4) they are flanked at their 5’ and 3’ ends by target site duplications (TSDs) of 5 to 20 nucleotides. The cDNA copies of mRNAs, the source of PPs, are inserted in far-flung regions of the genome [5]. At least 10% of PPs retain activity because when dispersed they have fortuitously landed close to an RNA polymerase II promoter [2]. We have known for ten years that the sequence characteristics of PPs are signs of mobilization by the endonuclease and reverse transcriptase activities of active LINE-1 (L1) elements [6,7]. In human cells, L1s have been shown to mobilize SINEs such as Alus [8,9], SVAs [10,11], and small nuclear (sn) RNAs [12], along with many mRNA transcripts. In mouse cells, L1s also mobilize B1 and B2 SINE elements [13]. More than 2,075 human genes are represented by at least one PP in the genome, while some genes, such as *GAPDH*, ribosomal proteins and actin β have 50 to 100 PPs [14]. Why 10% of human genes are represented by PPs, while the remaining 90% are not, is an important unanswered question.

A number of quite interesting PPs have been identified. In one example, the phosphoglycerate kinase gene, *pgk2*, is an active testis-expressed PP derived from the X-linked *pgk1* gene [15]. Deficiency of pgk2 leads to severe reduction in male fertility [16]. Another example is the fgf4 (fibroblast growth factor 4) PP in a number of dog breeds. This activated fgf4 PP is responsible for a chondrodysplasia that leads to the short-legged phenotype of 19 dog breeds, including dachsund, basset hound and corgi [17]. A third example is the *CypA* pseudogene that has inserted into the *TRIM5* gene at least twice, once in the owl monkey [18] and another time in the macaque lineage [19,20]. The *TRIM-Cyp* fusion gene leads to HIV-1 resistance of the monkeys because the TRIM-Cyp fusion protein blocks entry of the virus into cells [18].

There is another class of PPs termed semi-processed pseudogenes, which retain some introns and are particularly prevalent in the mouse and rat. For example, in the mouse the preproinsulin II gene has two introns, while the preproinsulin I gene is a PP that retains one of the two introns [21]. However, until very recently the prevailing view has been that there is very little ongoing PP formation in mammals. Now we know that that view is wrong. There is significant PP formation in present day human beings.

### Recent processed pseudogene insertions

**Figure 1:**
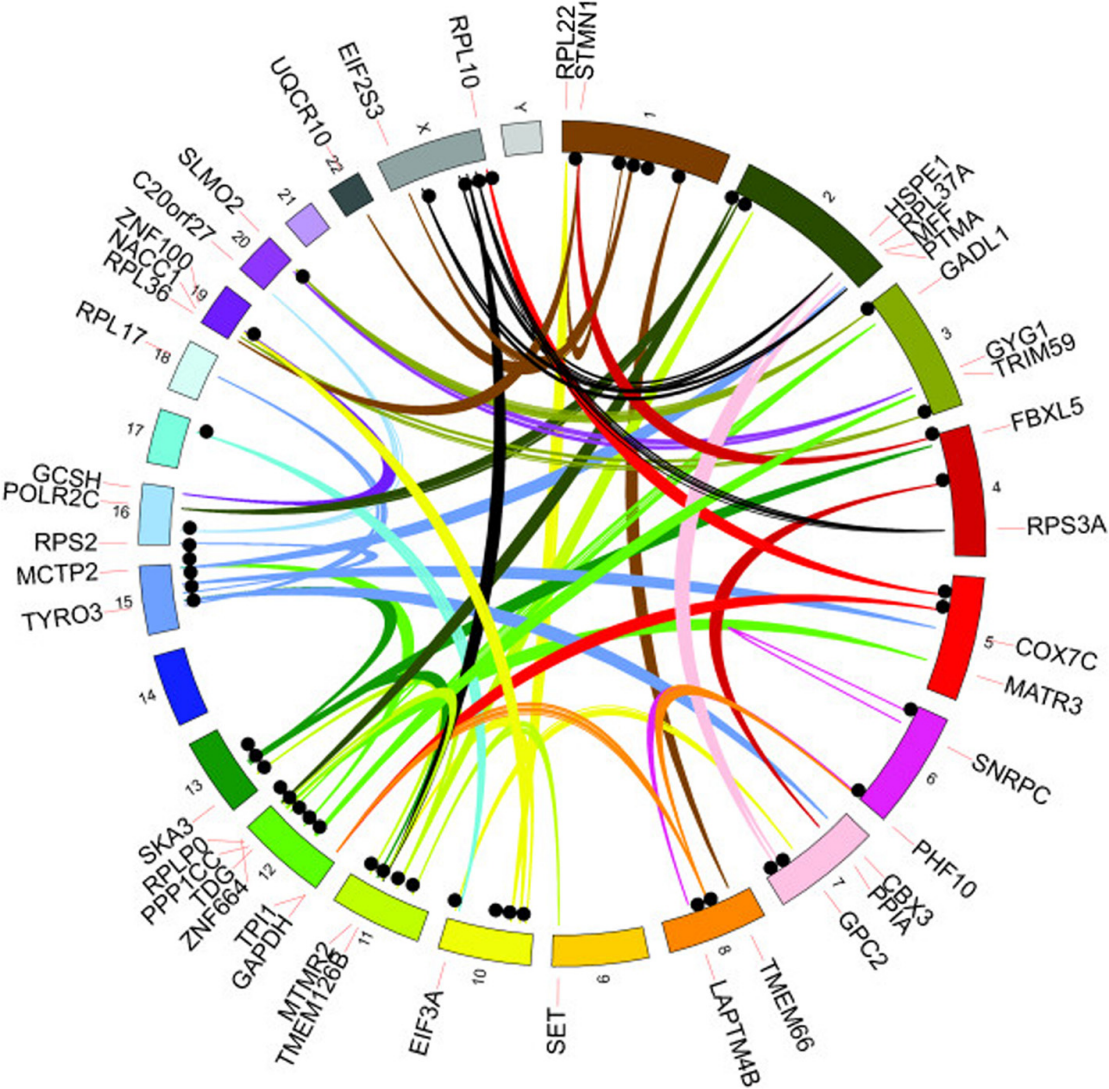
**Locations of 48 non-reference gene processed pseudogene insertions sites in the human genome based on reads mapped to source genes.** Discordant read mappings are represented by links colored based on chromosome of the source gene. Insertion sites are represented by black circles and the gene labels are based on the position of the source gene. Republished with permission from *Nature Communications*.

About one year ago, a comprehensive paper on polymorphism among PPs in human beings appeared. Ewing *et al*. devised a bioinformatic pipeline to detect polymorphic PPs. Using discordant reads not present in reference genomes, they found 48 novel PP insertion sites among 939 low pass genomes from the 1,000 genomes project [22]. These PPs came from a wide variety of source genes, and were spread throughout the human chromosomes (Figure 1). All 48 of these polymorphic PPs were confirmed by locating the precise genomic insertion site. This group also studied the genome sequences of 85 human cancer-normal tissue pairs representing a variety of cancers. Among these cancers they found the first instances of somatic insertion of PPs; three PPs were predicted to occur in lung cancers that were absent from paired normal tissue. The authors also estimated the rate of PP insertion in human beings at one insertion in every approximately 5,200 individuals/generation [22].

Ewing *et al*. went on to study PP polymorphism among mice, finding 755 new polymorphic PPs with most PPs occurring in species and subspecies derived from wild mice. Among these, *Mus musculus castaneus*, *M.m. musculus*, and *M.m. spretus* had 213, 212 and 142 PPs in their genomes, respectively, that were not found in the inbred C57Bl6 genome. However, on average, each of the 12 inbred strains derived from C57Bl6 were genetically closer, but still differed from one another by 68 PPs on average. The much greater number of polymorphic PPs in mouse strains compared to individual human beings may be due to the much larger number of active L1s present in the mouse (approximately 3,000 versus approximately 100 in humans) [23,24]. Ewing *et al*. also studied the genome sequences of ten chimpanzees and found ten polymorphic PPs among these animals. This paper represented the first comprehensive look at the question of PP insertions in humans, mice and chimpanzees, and the first study of somatic insertion of PPs in cancer.

Two other papers demonstrating polymorphism of PPs in humans have now appeared. Using exon-exon junction spanning reads, Abyzov *et al*. found 147 novel putative processed pseudogenes among approximately 1,000 low–pass genome sequences [25]. Thirty-six of these 147 were confirmed as polymorphic in humans by detection of the genomic insertion point. Interestingly, the parental genes of non-reference PPs were significantly enriched among genes expressed at the M-to-G1 transition in the cell cycle. Schrider *et al*. also mapped processed pseudogenes among 17 individuals, mostly using exon-exon junction spanning reads from SOLID and 1,000 genomes data [26]. They found 21 PPs not present in the reference genome and presumably polymorphic; 17 of these 21 were confirmed by PCR (See [27] for a recent review of these papers).

Recently, Cooke *et al*. studied somatic PP insertion in cancer in greater detail [28]. They analyzed 660 cancer-normal pairs of sequenced samples at Wellcome Trust representing a variety of different cancers. In 17 or 2.5% of the cancers, they found 42 somatic PPs. The authors noted the presence of five PPs in non-small cell lung cancer among 27 cancers studied, similar to the Ewing *et al*. finding of somatic PPs in lung cancer. Additionally, they found two PPs in eleven colorectal cancer samples.

The PP insertions in cancer were thoroughly characterized and all had the molecular signatures of germ line L1 insertions. The majority had TSDs of 5 to 20 base pairs, 74% were 5’ truncated (a percentage similar to that of human-specific L1s), 20% had inversions at their 5’ ends due to ‘twin priming’ (again similar to the rate in germ line human L1 insertions) [29], and long poly A tracts. In a lung adenocarcinoma, one insertion was associated with an 8 kb deletion of the promoter and exon 1 of a tumor suppressor gene, *MGA1*. The deletion knocked out expression of that allele as determined by RNA-seq.

Among the PPs in cancer, most were derived from highly expressed transcripts, yet many were not. In addition, many PP insertions appeared to be early events in tumor formation, being present in an early lesion along with the tumor or in multiple sections of the same tumor. However, some PP insertions were shown to be later events in tumor progression because they were not detected in all sections of the same tumor.

A final paper nailed down the potential for PP formation during early development in humans. This paper by de Boer *et al*. described a case of the X-linked disorder, chronic granulomatous disease in a Dutch man [30]. This man, now a young adult, had suffered from multiple bouts of pulmonary aspergillosis as a child. On workup of his *CYBB* (cytochrome b-245, beta polypeptide) gene, the defective gene in the disorder and parenthetically the first human gene cloned by positional cloning [31], it was discovered that a PP insertion had knocked out the gene’s activity.

**Figure 2:**
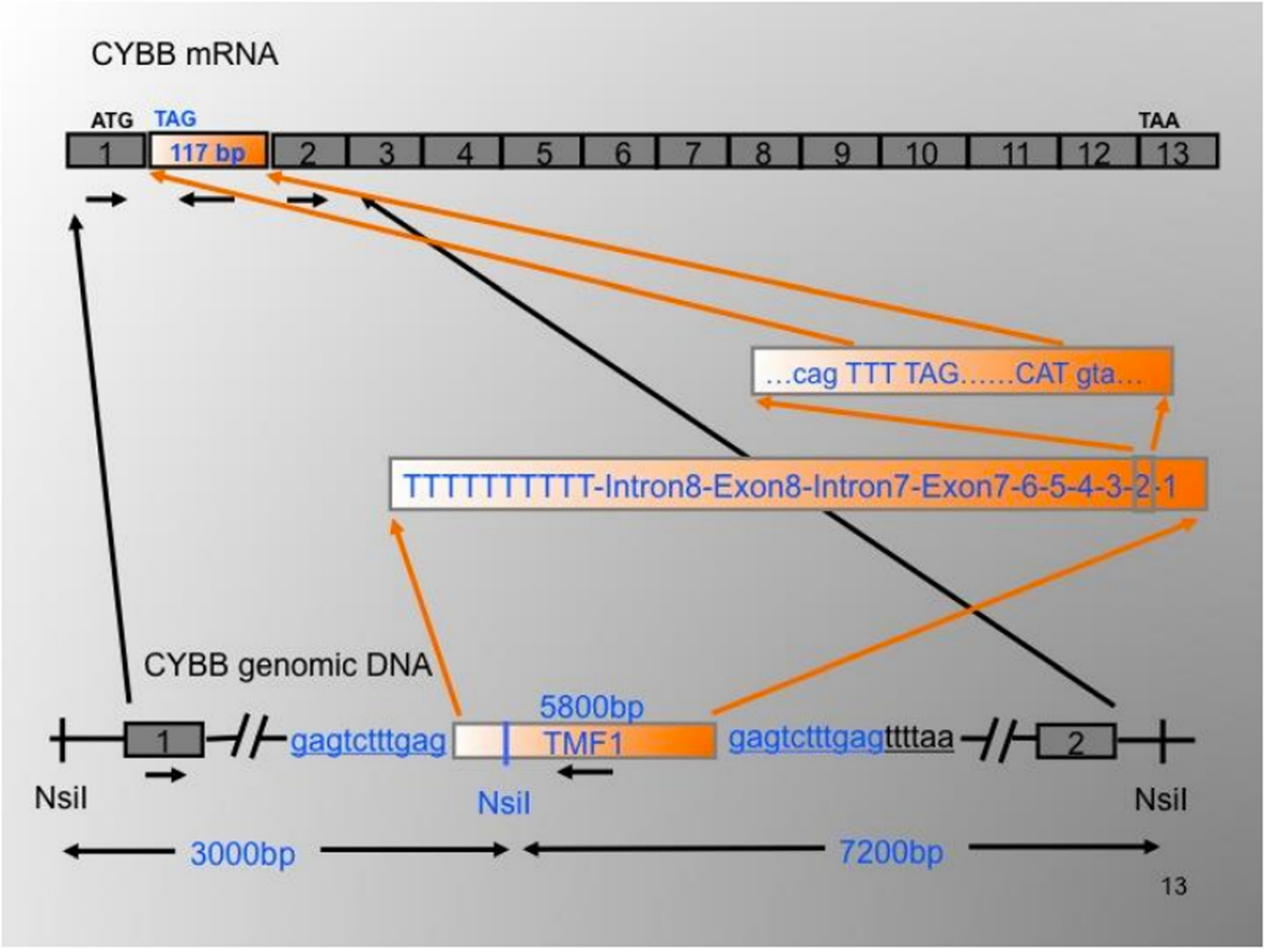
**Orientation of the TMF1 insertion in intron 1 of the *CyBB *gene (below), leading to an extra exon between exons 1 and 2 in the CYBB mRNA (above).** Republished with permission from *Human Mutation* published by Wiley.

There are three interesting aspects of this case. First, the insertion was a semi-processed pseudogene of the *TMF1* (TATA element modulatory factor) gene from chromosome 3 that had inserted into intron 1 of CYBB in reverse orientation. A PP had not been observed previously as a new insertion among 100 previous insertions (L1, Alu, SVA) in human Mendelian disease or cancer etiology [32]. Interestingly, *TMF1* is one of the about 10% of human genes that is represented by a single PP in the human reference genome sequence [14]. Second, the insertion was 3’ truncated and contained exons 1 to 8 of *TMF1* along with intron 7 and much of intron 8. Transcription of *TMF1* had terminated after an alternative poly A signal, AGUAAA, in intron 8, and a 100 bp poly A tail was added to the transcript. After insertion of this semi-processed pseudogene in reverse orientation into intron 1 of *CYBB*, splicing had occurred into an excellent acceptor splice site and out of an excellent donor site in exon 2 of TMF1. The newly created 117 bp exon also contained a nonsense codon that caused the *CYBB* gene to be non-functional (Figure 2). Finally, the PP insertion had occurred during early embryonic development of the patient’s mother. Roughly 10% to 20% of her lymphocytes contained the insertion as shown by qPCR.

To date, somatic retrotransposition in Mendelian disease has been rarely found. Among the 100 cases mentioned above, there is only a somatic insertion into the adenomatous polyposis coli (APC) tumor suppressor gene in a colorectal cancer case [33] and somatic and germ line mosaicism in the mother of a patient with the X-linked disease, choroideremia [34]. Thus, after more than 20 years since the discovery of the first retrotransposition events due to L1 and Alu elements [35,36], we finally have definitive evidence of retrotransposition of processed pseudogenes in human somatic cells (cancer and early development).

These papers beg the question, why do PP insertions not occur more frequently? Another recent paper has provided evidence that the RNAs associated with the L1 ORF1 protein in the L1 ribonucleoprotein particle (L1 RNP) contain a preponderance of those mRNAs that form PPs [37]. These mRNAs also have a much greater capacity for reverse transcription by L1 ORF2 protein than mRNAs that do not form PPs [37,38]. Now that we know that PP formation can occur in somatic cells, it is logical that those mRNAs that are both located in L1 RNPs and capable of reverse transcription have the inside track in PP formation. Messenger RNAs that lack what it takes to associate with the L1 RNP and be reverse transcribed, perhaps due to deficient cellular concentration or their sequence characteristics, are unable to form PPs. However, the story is not quite so simple since the majority of mRNAs that have formed PPs in the human genome do not appear to be associated with the L1 RNP. Thus, the demonstration of somatic PP insertions leads to a new as yet unanswered question: What are the important factors that increase the likelihood that a particular mRNA will become a processed pseudogene?

## Conclusions

Although perhaps unexpected, the evidence is overwhelming that PPs continue to insert in the germ line and in somatic cells of human beings.

## Abbreviations

PP: processed pseudogene; L1: LINE1-long interspersed element; RNP: ribonucleoprotein particle.

## Competing interests

The author declares that he has no competing interests.

## Authors’ contributions

HHK conceived and wrote the manuscript.
